# Contributions of biosurfactants to natural or induced bioremediation

**DOI:** 10.1007/s00253-013-4740-1

**Published:** 2013-02-12

**Authors:** Łukasz Ławniczak, Roman Marecik, Łukasz Chrzanowski

**Affiliations:** 1 Institute of Chemical Technology and Engineering, Poznan University of Technology, Pl. M. Skłodowskiej-Curie 2, 60-965 Poznań, Poland; 2 Department of Biotechnology and Food Microbiology, University of Life Sciences in Poznań, Wojska Polskiego 48, 60-627 Poznań, Poland

**Keywords:** Bioaugmentation, Biodegradation, Bioremediation, Biosurfactants, Phytoextraction

## Abstract

The number of studies dedicated to evaluating the influence of biosurfactants on bioremediation efficiency is constantly growing. Although significant progress regarding the explanation of mechanisms behind biosurfactant-induced effects could be observed, there are still many factors which are not sufficiently elucidated. This corresponds to the fact that although positive influence of biosurfactants is often reported, there are also numerous cases where no or negative effect was observed. This review summarizes the recent finding in the field of biosurfactant-amended bioremediation, focusing mainly on a critical approach towards potential limitations and causes of failure while investigating the effects of biosurfactants on the efficiency of biodegradation and phytoextraction processes. It also provides a summary of successive steps, which should be taken into consideration when designing biosurfactant-related treatment processes.

## Introduction

Surface-active compounds of biological origin have attracted much attention and their popularity seems to steadily increase during recent years. They are a frequent object of study, as the number of publications dedicated to the isolation and subsequent characterization of novel biosurfactant-producers is constantly growing (Ferhat et al. 2011; Shavandi et al. 2011; Zheng et al. 2012; Luna et al. 2013). This fact may be attributed to an evolved approach towards industrial production, which favors both environmental awareness and sustainability through use of renewable resources (Mukherjee and Das 2010). On the other hand, the fact that biosurfactants are characterized by a vast structural diversity and display a broad range of properties may also explain why this group of molecules continues to entice scientific curiosity (Marchant and Banat 2012). The numerous advantages of biosurfactants compared to their synthetic counterparts are yet another reason why these compounds seem so promising (Makkar and Rockne 2003; Soberón-Chávez and Maier 2011). While biosurfactants are generally equally effective in terms of solubilization and emulsification, they are also considered to be biodegradable, less toxic, and thus by far, more environmentally friendly than synthetic surfactants (Mulligan 2009). Since these molecules may be obtained from waste materials, their production also seems to be feasible in terms of economical justification (Mukherjee et al. 2006). All these relevant traits contribute to a high applicability of biosurfactants, which currently stems to several branches of industry (i.e., agriculture, cosmetics, food additives, and pharmaceutics; Muthusamy et al. 2008; Banat et al. 2010).

The much extolled environmental friendliness combined with the ability to solubilize hydrophobic compounds may well explain why biosurfactants have also been recognized as excellent agents for improving bioremediation of contaminated environments (Kosaric 2001). First and foremost, biosurfactants tend to interact with poorly soluble contaminants and improve their transfer into the aqueous phase. This allows for mobilization of recalcitrant pollutants which have been embedded in the soil matrix and their subsequent removal (Lai et al. 2009). The presence of biosurfactants may also lead to a potential enhancement of biodegradation efficiency. In this concept, the biosurfactant molecules act as mediators, which increase the mass transfer rate by making hydrophobic pollutants more bioavailable for microorganisms (Inakollu et al. 2004; Whang et al. 2009). Alternatively, biosurfactants may also induce changes in the properties of cellular membranes, resulting in increased microbial adherence. This mechanism is of importance when two immiscible phases (oil and water) are present and direct substrate uptake is plausible (Neu 1996; Franzetti et al. 2009). Another notable environmental application of biosurfactants is based on their ability to complex heavy metal ions, which may improve their removal or extraction via biological treatment (Mulligan et al. 1999, 2001).

Although the application of biosurfactants in bioremediation has been believed to be highly beneficial, soon several flaws and limitations have been revealed while testing the theories in practice. While potential enhancement has been achieved during initial short-term studies, no effect or even retardation has often been observed, especially for *in situ* treatment. The emerging contradiction may be explained by a wide lack of consistency between studies performed under laboratory conditions and practical environmental clean-up attempts.

The above-mentioned inconsistency regarding the actual efficiency of biosurfactants in bioremediation is the driving force behind this manuscript, which is focused on providing a critical overview of recent advances in biosurfactant-related studies. The aim of this mini-review is to plot the development in the field of biosurfactant-mediated bioremediation, cover the techniques where such compounds have found considerable usefulness, clearly summarize the findings in order to select crucial factors influencing their performance, highlight the causes of failures during biosurfactant supplementation studies, and outline the major considerations as well as possible restrictions regarding the applicability of these compounds for enhanced treatment purposes.

## The role of biosurfactants in bioremediation

### Definition of biosurfactants

Biosurfactants make for a peculiar group of compounds which exhibit notable distinction in terms of chemical structure and composition (Ron and Rosenberg 2001) and due to this fact they have found numerous interesting applications. The term “biosurfactants” is commonly associated with several different classes of molecules, such as glycolipids, lipopeptides, lipoproteins, phospholipids, fatty acids, as well as complex biopolymers (Rahman and Gakpe 2008; Mukherjee and Das 2010). Certain sub-classes have also been distinguished, some of which have become notably more popular than others. A prime example of this principle are rhamnolipids, an extensively studied and reviewed group of compounds (Soberón-Chávez et al. 2005; Abdel-Mawgoud et al. 2010), which often serves as a model biosurfactant for scientific experiments (Rahman et al. 2002; Górna et al. 2011). Regardless of the parent class all biosurfactants share a similar trait, namely their amphiphilic properties. Generally, biosurfactants exist either in an anionic or non-ionic form, however in most cases both the hydrophilic and the lipophilic part may be distinguished with relative ease. This particular characteristic is essential in terms of their contribution to bioremediation processes.

### Biosurfactants’ contribution to bioremediation

The main issue which directly influences the efficiency of biological treatment is the “bioavailability” of the pollutant. Possible sorption of molecules into the soil matrix, formation of non-aqueous phases, interactions with organic matter, biotransformation, and contaminant aging—these naturally occurring processes often result in limited bioavailability, thus decreasing the efficiency of bioremediation (Allard and Neilson 1997). The most common intended role of biosurfactants is therefore enhancing the distribution of contaminants into the aqueous phase and increasing their bioavailability.

As amphiphiles, biosurfactant exhibit the tendency to deposit at the oil/water interface. Biosurfactants may facilitate the transport of hydrophobic contaminants (i.e., hydrocarbon-based substances) into the aqueous phase through specific interaction resulting in solubilization and micellization (Costa et al. 2010). Increased mobilization allows for subsequent removal of such pollutants either by soil flushing or potentially makes them more susceptible to biodegradation (Maier and Soberón-Chávez 2000). Additionally, since heteroatoms are commonly present in the structure of biosurfactants, there are several active chemical groups (such as hydroxyl, carbonyl, or amine), which participate in the process of forming complexes with heavy metal ions. This process enables removal of heavy metal ions and may enhance their extraction efficiency using biological methods (Ochoa-Loza et al. 2001; Aşçi et al. 2008).

Apart from interactions with the pollutants the biosurfactants may also directly influence the efficiency of the corresponding bioremediator (microorganisms or plants), which is used for bioremediation. Biosurfactants exhibit strong biological activity, especially at the cellular membrane level. These modifications may result in enhanced hydrophobicity, which is considered to be relevant in terms of biodegradation efficiency, or change the permeability of cellular membranes, which would potentially be beneficial during bioextraction (Johnsen and Karlson 2004). However it has been established that the changes in cellular properties may not necessarily be associated with the ability to utilize certain carbon sources (Chakraborty et al. 2010), and therefore may not be easily correlated with bioremediation efficiency. For this reason, this topic will not be expanded in the framework of this review. For more information please refer to an excellent summary by Abbasnezhad et al. (2011).

Regarding the actual application of biosurfactants in bioremediation processes—the molecules may either be added externally (i.e., influent, spraying, injection) or produced on-site, which seems especially promising in case of *in situ* treatment. In the latter case, the production of biosurfactants may be obtained by bioaugmentation with appropriate microorganisms, since autochthonic microorganisms rarely exhibit satisfying efficiency.

### Effects of biosurfactants-supplementation on bioremediation efficiency

An overview of recent studies on biosurfactant-assisted bioremediation was presented in Table 1. It can be observed that although numerous studies reported a positive influence of biosurfactants on the bioremediation efficiency, there are also several cases where no effect or negative impact also occurred. Occasionally both positive and negative effects were noted, depending on the applied concentration. The prevalent use of rhamnolipids is worth noticing, as well as the fact that most frequently biosurfactants are introduced externally. These observations will be further elucidated and discussed in the next sections, which cover the use of biosurfactants during biodegradation and phytoextraction in detail.

**Table 1:** An overview of recent studies on biosurfactant-assisted bioremediation

Type of biosurfactant	Pollutant	Relevant bioremediator	Established effect	Removal efficiency	Reference
Rhamnolipids	Phenanthrene	*Sphingomonas* sp. monoculture	Positive—solublization	99 % after 10 days compared to 84 % without biosurfactant (IC—10 g/l)	Pei et al. (2010)
Rhamnolipids	Anthracene	*Sphingomonas* sp. and *Pseudomonas* sp. monocultures	Positive—solubilization	52 % after 18 days compared to 32 % without biosurfactant for *Pseudomonas* (IC—25 mg/l)	Cui et al. (2008)
Rhamnolipids (Mono-rhamnolipid)	Hexadecane	*Candida tropicalis* monoculture	Positive/negative	93 % after 4 days compared to 78 % without biosurfactants (IC—500 mg/l)	Zeng et al. (2011)
Rhamnolipids, emulsan and indigenous biosurfactants	Pyrene	*Pseudomonas fluorescens* monoculture	Positive/negative	98 % after 10 days compared to 91 % without emulsan (IC—50 mg/l)	Husain (2008)
Rhamnolipids	Polycyclic aromatic hydrocarbons	Alfalfa + arbuscular mycorrhizal fungi + microbial consortium of PAH degraders	Positive—solubilization	61 % after 90 days compared to 17 % with only phytoremediation (IC—12.85 g/kg of soil)	Zhang et al. (2010)
Rhamnolipids (Mono-rhamnolipid)	Phenol	*Candida tropicalis* monoculture	Positive—enhanced cell growth	99 % after 30 h compared to 87 % without biosurfactant (IC—500 mg/l)	Liu et al. (2010)
Rhamnolipids	Crude oil hydrocarbons	Autochthonous marine microflora	Positive/no effect	Up to 25 % for alkanes after 5 days with biosurfactant alone and 59 % when used with nutrients (IC—823 mg/l)	McKew et al. (2007)
Rhamnolipids	Crude oil hydrocarbons	Autochthonous marine microflora	Positive—increased bioavailability	96 % for C19–C34 alkane fraction after 18 days compared to 10 % without amendment (IC—5 g/l)	Nikolopoulou and Kalogerakis (2008)
Rhamnolipids	Phenanthrene	*Sphingomonas* sp. and *Paenibacillus* sp. monocultures	Negative	23 % after 8 days compared to 74 % without biosurfactant	Shin et al. (2005)
Rhamnolipids	Phenanthrene	*Pseudomonas putida* ATCC 17484 monoculture	Positive/no effect/negative	91 % after 10 days compared to 68 % without biosurfactant (IC—approx. 500 mg/kg of soil)	Gottfried et al. (2010)
Rhamnolipids	Phenanthrene	*Sphingomonas* sp. monoculture	Positive—mobilization	47 % after 70 days compared to 36 % without biosurfactant (IC—approx. 200 mg/kg of soil)	Shin et al. (2006)
Rhamnolipids	Diesel oil and biodiesel blends	Microbial consortium	Positive/no effect	77 % after 7 days compared to 58 % without biosurfactants for blends (IC—approx. 15 g/l)	Owsianiak et al. (2009a, b)
Rhamnolipids	Phenanthrene and pyrene	Ryegrass	Positive—increased uptake	Uptake of phenanthrene and pyrene into ryegrass roots was at 435 and 380 mg/kg, respectively, compared to 77 and 158 mg/kg without biosurfactant	Zhu and Zhang (2008)
Rhamnolipids	Cadmium	*Vibrio fischeri*, *Pseudomonas fluorescens*, *P*. *aeruginosa*, *Escherichia coli* and *Bacillus subtilis* monoluctures	Positive/negative	Rhamnolipids were toxic at higher concentrations (>45 mg/l), however at 40 mg/l their presence inhibited the toxicity of cadmium ions by reducing their bioavailability	Bondarenko et al. (2010)
Rhamnolipids and organic acids	Copper	Indian mustard and ryegrass	Positive—mobilization	Application of rhamnolipids and other amendments notably increased copper uptake by both plants	Johnson et al. (2009)
Sophorolipid	Hydrocarbon mixture	Autochthonous soil microflora	Positive—solubilization and mobilization	Respectively: 95 % after 2 days, 97 % after 6 days and 85 % after 6 days (IC- 6 mg/g of soil)	Kang et al. 2010
Not specified	p,p'-DDE	*Cucurbita* subspecies	Positive/negative	Biosurfactant amendment enhanced p,p′-DDE accumulation, however a 60 % biomass reduction was observed for *ovifera* subspecies	White et al. (2006)
Not specified	Petrochemical oily sludge	Mixed bacterial cultures	Positive—potential solubilization	91 % of the aliphatic fraction and 52 % of the aromatic fraction after 40 days	Cerqueira et al. (2011)
Not specified	Diesel oil hydrocarbons	Autochthonous soil microflora	Positive/no effect	77 % of aliphatic hydrocarbons after 15 days compared to 9 % without biosurfactant (IC—450 mg/l)	Martins et al. (2009)
Not specified	Pyrene	*Bacillus subtilis* and *Pseudomonas aeruginosa* monocultures	Positive—solubilization	48 % for *Bacillus* and 32 % for *Pseudomonas* after 4 days	Das and Mukherjee (2007)

*IC* initial concentration at the start of the experiment

## Application of biosurfactants during biodegradation of xenobiotics

### Impact of biosurfactants on bioavailability of pollutants

A notable number of previous studies analyzed the influence of biosurfactants on biodegradation processes mainly in terms of efficiency enhancement. Potential stimulation was mostly associated with solubilization of pollutants, resulting in their increased bioavailability. For example Moldes et al. (2011) carried out studies focused on assessing the influence of biosurfactants from *Lactobacillus pentosus* on the biodegradation efficiency of octane in soil by autochthonous microflora. After 15 days, the biodegradation efficiency reached 59 % and 63 % for soil contaminated with 700 and 70,000 mg/kg of octane in the presence of biosurfactants, while in their absence the removal rate was at 1 % and 24 %, accordingly. The authors suggested that mobilization of octane molecules and subsequent increase in their bioavailability was the main cause of the observed differences. The results obtained by Manickam et al. (2012) also confirm that biosurfactant-supplementation is also a feasible strategy for enhancing the biodegradation of halogenated compounds. It was observed that the biodegradation efficiency for all biosurfactant-amended samples (rhamnolipids, sophorolipids, or trehalose lipids) was increased by 30–50 % in 2 days compared to degradation after 10 days in the absence of surfactant. This was true for both batch culture experiments and spiked soil slurry studies.

It has also been recognized that in addition to mobilization, the biosurfactants may also enhance biodegradation efficiency by other mechanisms. An interesting form of interactions between biosurfactants and toxic contaminants was presented by Chrzanowski et al. (2009), where rhamnolipids were used as agents which reduce the toxicity of chlorinated phenol homologues towards monoculture of *Pseudomonas putida* DOT-T1E. This phenomenon was further elucidated in Chrzanowski et al. (2011) during studies on biodegradation of a hydrocarbon-rich petroleum effluent by a microbial consortium in the presence of chlorophenols. Due to entrapment of chlorophenols in biosurfactant micelles as well as hydrophobic interactions between these two groups of compounds, the toxicity of phenol-based molecules could be substantially reduced. This in turn resulted in increased microbial growth and enhanced biodegradation of hydrocarbons present in the petroleum effluent. Other studies also confirm that the addition of rhamnolipids may improve the biodegradation of petrochemical industry wastewater (Sponza and Gok 2011).

### Combined supplementation with biosurfactants and additional amendments

Currently much emphasis is directed towards properly addressing the corresponding environmental factors and recognizing the involved mechanisms. Recent findings have clearly confirmed that even if the availability of carbon sources is high, the microbial growth will still be inhibited when the concentration of relevant microelements is limited. As a result, the biosurfactant-amendment is now frequently combined with the addition of nutrients. For example, Cameotra and Singh studied the effect of crude biosurfactants and nutrient amendment on the biodegradation of oil sludges of different origin carried out by a mixed culture (two *Pseudomonas aeruginosa* strains and one *Rhodococcus* sp. strain) in soil (Cameotra and Singh 2008). A notable difference in terms of biodegradation efficiency was observed upon the addition of biosurfactants and nutrients during experiments compared to the inoculation with the mixed culture without any additives (removal at 98 % and 52 % after 8 weeks, respectively). The amendment with a combination of both additives proved more efficient compared to samples where only biosurfactants (removal at 73 %) or nutrients (removal at 63 %) were added to the inoculated oil sludge. Similar results regarding the efficiency of combined amendment with biosurfactants produced by *Lactobacillus delbrueckii* and fertilizer were reported by Thavasi et al. (2011a, b). These results stress out that apart from bioavailability issues, a sufficient amount of crucial nutrients, such as nitrogen or phosphorous, is also a key factor for an efficient bioremediation process.

### Influence of biosurfactants on the degrading microorganisms

Interestingly, Bordoloi and Konwar reported that biosurfactants obtained from different *P. aeruginosa* strains favored specific petroleum hydrocarbons in terms of enhanced solubility and metabolism (Bordoloi and Konwar 2009). While some of the isolated biosurfactants caused increased solubility of pyrene, other contributed to a higher solubilization rate of phenanthrene or fluorene. These differences were also notable during short-term tests regarding the reduction of crude oil, phenanthrene, pyrene, and fluorene from the culture medium. Overall, the uptake of each of the tested hydrocarbons was significantly increased in all bacterial cultures upon the addition of biosurfactant.

A recent study regarding the effect of biosurfactant and fertilizer amendment on the biodegradation of crude oil by marine isolates of *Bacillus megaterium*, *Corynebacterium kutscheri* and *P. aeruginosa* was carried out by Thavasi et al. (2011a, b). The experiments were conducted in flasks and laboratory scale microcosm with natural sea water. During the microcosm experiments, a considerable difference in the crude oil degradation efficiency among the studied isolates could be observed upon amendment. While the changes were not significant for *Corynebacterium kutscheri* and *B. megaterium*, the introduction of either biosurfactants or fertilizer into samples with *P. aeruginosa* cells greatly enhanced the crude oil biodegradation rate. The best results were obtained when both additives were introduced (approx. 90 % removal) compared to samples without any amendment (approx. 50 % removal). The results of this study, combined with the previous report, lead to the conclusion that in some cases the use of biosurfactants may contribute to substrate- or species-specific changes in the biodegradation efficiency.

The latter statement leads to a discussion regarding the efficiency of biosurfactant-amendment in relation to the behavior of microorganisms participating in the treatment process. It was often observed that the application of biosurfactants at higher concentrations may inhibit the microbial growth and thus decrease the biodegradation efficiency. This was reported by Whang et al. (2008) during studies focused on biosurfactant-mediated biodegradation of diesel-contaminated water and soil carried out by autochthonic soil microorganisms during batch diesel/water experiments and biopile tests. The authors observed that even though diesel solubilization was slightly higher for surfactin, especially above the CMC value, the presence of this surfactant may limit the biodegradation rate at concentrations above 40 mg/l (with a complete inhibition at 400 mg/l). Since the biomass growth was also inhibited, the preferential utilization of surfactant was excluded and possible toxicity issues seemed more plausible. As described above, the potential toxicity of biosurfactants towards microbes at higher concentrations may be an issue affecting their applicability. The nature of this topic is not unequivocal, since some reports show that the toxicity of biosurfactants is low (Lima et al. 2011a, b), while other studies prove that such compounds often exhibit antimicrobial properties (Vatsa et al. 2010). Although it is commonly considered that biosurfactants are non-toxic at low concentrations, there question which follows is whether such concentrations may be of relevance during bioremediation? The problem is even more challenging when considering the biodegradation of polluted soil, since sorption of biosurfactants into the soil matrix would decrease their effective concentration.

### Biosurfactants and microbial consortia

It should be pointed out that the majority of the previous studies on biosurfactant-mediated biodegradation were carried out with the use of monocultures. On rare occasions, mixed cultures were used; however currently more emphasis is directed towards microbial consortia. Several recent studies prove that the use of consortia contributes to increased biodegradation efficiency compared to monocultures (Kadali et al. 2012), since the cooperation between the individual consortium members and the complementary effect of microbes on each other may result in notably enhanced growth and survivability (Sampath et al. 2012). The studies carried out by Owsianiak et al. (2009a, b) focused on evaluating the effect of rhamnolipids on the biodegradation potential of 218 bacterial consortia isolated from petroleum contaminated soil with respect to changes in cell surface properties. Overall, it was observed that the addition of biosurfactant increased the biodegradation efficiency for slow-degrading consortia, while a notable decrease of biodegradation rate occurred for fast degrading consortia. This phenomenon may potentially be explained by different substrate uptake modes. The slow-degrading consortia most likely preferred uptake of hydrocarbons from the aqueous phase, therefore solubilization of hydrocarbons enhanced the biodegradation. On the other hand, the consortia with a high initial biodegradation potential displayed the tendency to form biofilms on the interfacial boundary, which suggested that direct uptake mechanisms were favoured. As biosurfactants deposit on the oil–water interface, their presence would limit the contact between microorganisms and substrates and thus inhibit the biodegradation rate. In this scenario the biosurfactant layer would be an obstacle for microbial uptake of hydrocarbons and should therefore be removed in order to proceed with the biodegradation process. Since biosurfactants may potentially be biodegraded, the discussion will focus on this issue.

### Biodegradability of biosurfactants in relation to bioremediation efficiency

The biodegradability of biosurfactants has been unquestionably considered as their major merit. Several studies confirm that biosurfactants exhibit higher biodegradability compared to surfactants of synthetic origin (Lima et al. 2011a, b). It is true that this property makes them more promising, since they would not persist in the environment upon treatment. On the other hand it should be pointed out that biodegradability comes at the cost of process sustainability, as biosurfactants will be slowly removed and their effect will be diminished. The studies carried out by Lin et al. (2011) confirm that although the process efficiency was greatly enhanced by the addition of biosurfactants in the initial stage, the biodegradation rate in the latter stages was similar to that obtained during treatment in the absence of biosurfactants. It is also plausible that biosurfactants may be biodegraded before their expected action takes place. Either due to the above-mentioned issue of biosurfactants interfering with direct uptake of hydrocarbons or simply because of the fact that these molecules may be treated as an alternative carbon source—preferential utilization of biosurfactants compared to target contaminants is a highly negative pattern. Such case was recently described by Chrzanowski et al. (2012a, b). It was observed that rhamnolipids were preferentially biodegraded compared to diesel oil under both aerobic and anaerobic conditions. As a result no stimulation of hydrocarbon removal occurred compared to samples not amended with biosurfactants.

A possible solution to this problem includes the application of microorganisms which do not preferentially degrade biosurfactants. Several studies related to this topic suggest that this trait is often observed for biosurfactant producers (Providenti et al. 1995; Zeng et al. 2007). The studies carried out by Hidayati et al. (2011) confirmed that the external addition of a crude biosurfactant mixture produced by *B. megaterium* into samples inoculated with these microorganisms resulted in enhanced biodegradation efficiency of fluorine. Interesting results were obtained by Tzintzun-Camacho et al. (2012) during studies on the biodegradation efficiency of a microbial consortium in relation to the performance of each individual member. The highest biodegradation efficiency was observed when the whole consortium was used (79 %). However, the removal rate for samples inoculated solely with *Acinetobacter bouvetii*, the only bacterial taxa capable of producing biosurfactants, was similar (72 %) and much higher compared to the performance of other members. Saimmai et al. (2012) also observed that the biodegradation potential of a hydrocarbon-degrading consortium was correlated with its ability to produce biosurfactants. These observations suggest that the contribution of biosurfactant producers to the biodegradation process may be crucial.

### Current strategies regarding the introduction of biosurfactants in order to enhance the biodegradation efficiency

The application of a consortium, which consists of members capable of producing biosurfactants is a promising strategy, since possible sustainability may be achieved. With this in mind bioaugmentation attempts involving biosurfactant producers were carried out. Interestingly, while the procedure of inoculation with biosurfactant producers has earned a notable degree of applicability in microbial enhanced oil recovery technologies, the same cannot be said about biodegradation processes. In most cases no notable changes in the biodegradation efficiency were observed (Jain et al. 1992; Sun et al. 2012). Dean et al. (2001) observed that co-inoculation of a biosurfactant producing *P. aeruginosa* ATCC 9027 strain with two other strains of phenanthrene degraders resulted in fundamentally different effects. While no stimulation was observed in one case a notable increase of the biodegradation efficiency was observed in the other. This suggests that specie-specific interactions play a crucial role for successful bioaugmentation. It should be pointed out that recent advances in bioaugmentation approaches regarding proper strain selection, consideration towards environmental factors and microbial ecology, which have been elucidated in an excellent review by Thompson et al. (2005), are of especially great value in the field of bioremediation. Biodegradation is a process where competition for carbon sources often results in antagonistic interactions between microorganisms, therefore the odds of successfully introducing certain microbes into the polluted environment will be increased by conscious selection. A particularly interesting approach involves the isolation of autochthonic microbes, genetic engineering aimed at introducing biosurfactant production genes and re-introduction of the recombinants into the polluted area, however at this moment the number of studies which would verify the feasibility of this strategy is very limited. Overall, since the current bioaugmentation protocols must adhere to strict regulations and do not ensure that the desired treatment efficiency will be achieved, this strategy is potentially promising yet rarely employed.

For this reason external addition of biosurfactants has become a common procedure in biosurfactant-amended bioremediation. The study carried out by Henry et al. (2011) focused on evaluating the effect of encapsuled biosurfactants on emulsification and biodegradation efficiency of phenanthrene. Such an approach may also potentially enhance the sustainability of biosurfactant-mediated biodegradation, since the relevant molecules would be constantly released throughout the process; however, the results showed that the performance of encapsuled biosurfactants was inferior to the non-encapsulated biosurfactants. The authors suggested that an immediate formation of emulsion was crucial in order to improve the biodegradation efficiency. Interesting results regarding the combined effect of bioaugmentation and biostimulation on the bioremediation efficiency of oil-contaminated soil were presented by Lin et al. (2010). The authors established that introduction of pre-selected microorganisms coupled with the addition of biosurfactants notably increased the TPH removal rate and substantially reduced the treatment duration, while the application of a molecular microarray biochip for monitoring ensured that the process is progressing in a satisfactory manner. This complex technology, labeled Systematic Environmental Molecular Bioremediation Technology (SEMBT), may be a potentially promising bioremediation strategy.

## Application of biosurfactants during phytoextraction of heavy metal ions

### Biosurfactant-induced changes in the mobility of heavy metal ions

Biosurfactant-assisted removal of heavy metal ions by complex formation and subsequent mobilization has received much attention. This method offers relatively high efficiency and reduced environmental hazardousness compared to flushing with synthetic surfactants. The studies carried out by Gao et al. (2012) regarding potential recovery of heavy metal ions in sludge from an industry water treatment plant by application of biosurfactants confirm that bio-based surface active compounds exhibit high selectivity towards certain heavy metal ions. The authors also observed that the type of biosurfactant may impact the removal efficiency, as the effect of saponins was found to be greater compared to sophorolipids. The results obtained by Lima et al. (2011a, b, c) imply that biosurfactants may be successfully used for simultaneous removal of heavy metal ions and organic pollutants. It was reported that the application of lipopeptides obtained from different bacterial strains notably enhanced the removal rate of cadmium (99 %) as well as phenanthrene (80–88 %).

The application of biosurfactants in phytoextraction (extraction with the use of plants) of heavy metal ions may potentially be beneficial; however, recent reports have also revealed certain limitations. The studies carried out by Gunawardana et al. (2010) focused on the influence of different amendments (aminopolycarboxylic acid–EDDS, histidine, citric acid, rhamnolipids, and sulfate) on the efficiency of copper, cadmium and lead uptake by *Lolium perenne* revealed an enhancement of phytoextraction. The combined use of EDDS, rhamnolipids, and citric acid contributed to a most notable translocation of metals to shoot tissue, however the authors observed that this amendment caused severe phytotoxicity. The studies carried out by Marecik et al. (2012) confirmed that the sole presence of rhamnolipids may cause a notable inhibition of the germination index and biomass gain for certain plant species. It was observed that sorghum was most susceptible, followed by alfalfa and mustard species, while cuckooflower exhibited the highest resistance. These results suggest that phytotoxicity of biosurfactants is specie-specific and should be taken into consideration when planning treatment processes. The actual applicability of rhamnolipids for enhancing phytoextraction efficiency was addressed by Wen et al. (2010). The experiment focused on rhamnolipids-amended extraction of cadmium from soil by maize and sunflower with regard to potential phytotoxicity. The authors established that the use of rhamnolipids at higher concentrations (>4.4 mmol/kg) resulted in severe phytotoxicity towards both plant species. On the other hand, the use of lower concentrations (0.02–1.4 mmol/kg) did not improve cadmium accumulation, most likely due to sorption of rhamnolipids into the soil matrix. Based on the obtained results, the authors established that neither high nor low concentration of rhamnolipids is likely to consistently assist cadmium phytoextraction using maize and sunflower.

Interesting results regarding the problem of biosurfactant biodegradation prior to their effect as well as potential issues associated with uncontrolled mobilization and spreading of pollutants were presented by Wen et al. (2009). It was observed that the biodegradation of rhamnolipids in cadmium and zinc contaminated soils was lower compared to uncontaminated soils, suggesting that due to specific interactions between metal ions and chelating agents during complex formation the biodegradability of surfactants may be influenced. The authors established that the applicability of rhamnolipids for mobilization of heavy metal ions is justified in terms of their biodegradability, since this biosurfactant persists long enough to enhance the extraction but is not recalcitrant and therefore should not contribute to uncontrolled transport of metal ions.

### Possible use of biosurfactant-producing microbes for enhanced phytoextraction

Although enhancement via bioaugmentation also seems like a promising strategy for biosurfactant-mediated phytoextraction, in this case the introduction of microorganisms possessing relevant genes is perhaps even more challenging compared to biodegradation. The limitations of bioaugmentation-assisted phytoextraction of heavy metal polluted soil have been discussed in an excellent review by Lebeau et al. (2008). Since the review is focused on highlighting recommendations regarding proper selection of microorganisms and factors influencing bioaugmentation, the authors point out the importance of assessing potential survivability and soil colonization abilities as crucial prerestiques. It was also stressed out that plant–bacteria associations are not easily modified and thus non-competence among the introduced bacteria and plants often results in failures of bioaugmentation attempts.

Overall, the selected microorganisms should exhibit tolerance towards high concentrations of heavy metal ions and high compatibility with plants used for phytoextraction. Unfortunately, these requirements are rarely met by conventional biosurfactant producers. Therefore the application of rhizosphere microbes (which exist in close proximity to plant roots) potentially offers higher odds of success, since it is considered that the metal-resistance of such microorganisms is approximately ten times greater compared to microbes originating from bulk soil (Lodewyckx et al. 2002). The studies carried out by Becerra-Castro et al. (2011) regarding solubilization of nickel by bacteria isolated from the rhizosphere of *Alyssum serpyllifolium* provide insight in terms of biosurfactant production in the rhizosphere. It was observed that out of 84 strains selected for studies only 13 were able to successfully mobilize nickel ions in soil. Similar observations were made by the same authors in a different study (Becerra-Castro et al. 2012), where 15 out of 74 rhizobacteria exhibited the ability to produce biosurfactants. Overall, biosurfactant producers accounted for 15–20 % of the total number of isolates. It is worth noticing that the authors established a lack of relation between the microbial ability to mobilize metal ions and tolerance towards such contaminants. This fact may explain why currently more emphasis is put into selection of metal-tolerant plant growth promoting microorganisms and the number of studies dedicated to introduction of biosurfactant producers is limited (Braud et al. 2006; Sheng et al. 2008).

## Relevant steps for designing biosurfactant-mediated bioremediation

Taking into consideration the above-mentioned reports, it can be concluded that bio-compatibility between each relevant treatment factor (pollutant, microorganisms/plants, and biosurfactants) is necessary to achieve efficient bioremediation. The corresponding environmental factors as well as the influence of native microflora should also be taken into consideration, when attempting to carry out *in situ* clean-up. The lack of clearly specified guidelines for the selection of a proper treatment approach contributes to a certain amount of randomness in designing the experiments, which often result in failure. Based on the lessons from the previous studies a series of successive steps was constructed in order to enhance the odds of successfully choosing the treatment factors for bioremediation in future studies (Table 2).

**Table 2:** Successive steps which should be taken into consideration during the design of biosurfactant-mediated bioremediation processes

Design step	Relevant step	Criteria
I. Initial characterization of the polluted area	1. Initial recognition of pollutants	Establishment of either single or multi-contaminant type pollution
	2. Assessment of the target pollutants concentration range	Determination of readily bioavailable, potentially bioavailable and unavailable pollutant fractions
	3. Analysis of relevant environmental factors	Range of temperature, pH, redox potential, moiety, soil properties, etc.
	4. Evaluation of nutrient levels	Potential limitation due to insufficient microelements, electron acceptors, etc.
	5. Analysis of autochthonous microflora	Screening for native microbial consortia with the ability to either remove or mobilize the pollutant by producing biosurfactants
II. Laboratory scale experiments	1. Selection of appropriate bioremediators for conducting the bioremediation process	Microorganisms or plants which exhibit high tolerance toward target pollutants and distinct remediation potential (relevant catabolic genes, hyperaccumulative properties, etc.)
	2. Selection of additional amendments	Nutrients, co-inoculants, plant growth promoting microorganisms, arbuscular mycorrhizal fungi, etc.
Laboratory scale feasibility studies for biosurfactant-supplementation, approach A: Addition of externally produced biosurfactants (*ex situ* methods)	1. Selection of a biosurfactant and biosurfactant-producing microorganisms	Previous studies related to the topic or the native habitat of biosurfactant-producing microorganisms
	2. Assessment of potential biosurfactant-induced toxicity	EC50 values for relevant bioremediators towards biosurfactant only as well as biosurfactant-pollutant combinations; Analysis of microbial community dynamics as a response to the presence of biosurfactants
	3. Evaluation of efficiency for biosurfactant-amended remediation	Increase in pollutant bioavailability, increased removal rate, short-term stimulation, enhanced biomass growth for the bioremediator
	4. Determination of biosurfactant degradability	Biosurfactant not preferentially utilized compared to target pollutant, efficient usefulness period for short-term stimulation, time for re-introduction
	5. Establishment of an optimal biosurfactant production method	Assessment of potential carbon sources for biosurfactant production (waste materials); optimization of the production process; Determination of whether crude biosurfactant-containing cultivation broth may be used or is purification necessary
Laboratory scale feasibility studies for biosurfactant-supplementation, approach B: Stimulation of biosurfactant production on-site (*in situ* methods)	1. Selection of appropriate biosurfactant-producers	Preferentially – selection of biosurfactant-producing isolates from native microflora (autochthonous soil/marine microbes, rhizobacteria, etc.); Alternatively – use of non-producing isolates which may be genetically modified to secrete biosurfactants or application of microbial consortia with high bioaugmentation potential (high similarity between consortium members and autochthonous microorganisms). Both alternative approaches are subject to additional regulations
	2. Evaluation of biocompatibility between biosurfactant producers and the biofactor relevant for the treatment process	Lack of antagonistic interactions, simultaneous growth, increase in pollutant bioavailability, enhanced removal rate
	3. Selection of an introduction method	Spraying of the whole cultivation broth with free-living cells or immobilization on appropriate carriers
	4. Initial bioaugmentation tests	Satisfactory performance in terms of adaptability and survivability of the introduced biosurfactant-producers, no apparent shifts in microbial community dynamics, lack of antagonistic interactions with native microflora
	5. Long-term ability to produce biosurfactants	Monitoring the level of biosurfactants upon bioaugmentation, the presence of relevant biosurfactant-associated genes after a certain period of time
III. Field scale feasibility study	1. Environmental response towards biosurfactants or biosurfactant-producers	Shifts in microbial populations; toxicity of biosurfactant to native organisms; adaptability and survivability of bioremediators and/or biosurfactant producers upon introduction; other potentially negative effects (i.e. uncontrolled mobilization of pollutants)
	2. Efficiency of treatment	Short-term and long-term removal of target pollutants in biosurfactant-amended treatment compared to control; duration
	3. Evaluation of treatment feasibility	Justification of each treatment step; Potential efficiency enhancement vs. additional costs associated with biosurfactant-supplementation

The initial characterization prerequisites mostly cover the common steps for each environmental clean-up approach, however much emphasis is directed toward analysis of native microflora. While natural attenuation is rarely efficient in terms of time, the recognition of most abundant taxa in the autochthonic populations may prove crucial for achieving success in the latter steps. The next step is associated with the selection of appropriate bioremediators, which will be relevant for the treatment process. Regardless of whether the process is focused on the application of bacteria, fungi or plants—their survivability and adaptation to the contaminated environment is of greatest importance. With this in mind, the chosen bioremediators should exhibit high similarity to the adequate native organisms. The subsequent stages are dedicated to the selection of an adequate biosurfactant introduction method—either the addition of externally produced biosurfactants (*ex situ* methods) or possible stimulation of biosurfactant production on-site (*in situ* methods). Taking into consideration the fact that bioaugmentation must follow strict regulations and the overall low feasibility associated with on-site production, the introduction of biosurfactants which are produced outside of the polluted area is currently considered as a more solid approach. Regardless of the chosen strategy, the selection of appropriate biosurfactants or biosurfactant-producing microorganisms along with placing priority towards enhancing the bio-compatibility and evaluating the optimal concentrations for the process (balanced approach which covers both toxicity and biodegradability of biosurfactants) should be considered as crucial factors. Finally, the treatment set-up which seems satisfactory under laboratory conditions should be tested in field conditions. This step ultimately provides an answer regarding the feasibility of the treatment process.

## Conclusions and future considerations

The application of biosurfactants in bioremediation processes is currently an ambiguous topic. Although undoubtedly positive influence in terms of pollutant removal efficiency was reported on several occasions, there are also numerous cases where no effect or even inhibition of removal rate was observed. The main reason is perhaps the inconsistency between the intended role of biosurfactants in contaminant treatment processes (increasing the bioavailability of pollutants) and their actual role in the ecology of microorganisms—which by far surpasses the boundaries of bioremediation (Tremblay et al. 2007; Glick et al. 2010; Chrzanowski et al. 2012a, b). However we believe that these two topics are closely related, since understanding the multiple contributions of biosurfactants to different aspects of microbial existence is crucial for their successful application in biological remediation. Future studies should not only concentrate on an efficiency-focused approach, but also on expanding this challenging problem by elucidating the complex interactions of biosurfactants, microorganisms, and pollutants.
